# 555 Pain Management During Bromelain-Based Enzymatic Debridement in a U.S. Adult Burn Center

**DOI:** 10.1093/jbcr/irad045.151

**Published:** 2023-05-15

**Authors:** Martin Buta, Domenic Annand, Sarah Findeisen, Sean Hickey, Robert Sheridan, Jonathan Friedstat, John Schulz, Branko Bojovic, Jeremy Goverman

**Affiliations:** Massachusetts General Hospital, Boston, Massachusetts; Massachusetts General Hospital, Boston, Massachusetts; Massachusetts General Hospital, Boston, Massachusetts; Massachusetts General Hospital, Boston, Massachusetts; Massachusetts General Hospital, Boston, Massachusetts; Massachusetts General Hospital, Boston, Massachusetts; Massachusetts General Hospital, Boston, Massachusetts; Massachusetts General Hospital, Boston, Massachusetts; Massachusetts General Hospital, Boston, Massachusetts

## Abstract

**Introduction:**

Outside the United States, bromelain-based enzymatic debridement (BBED) has become an effective tool for removal of burn eschar. BBED can be performed at bedside and allows for complete eschar removal with maximal dermal preservation and markedly reduced blood loss. It may also decrease the need for autografting or the amount of autograft required. A primary concern with BBED is that it is a painful procedure requiring appropriate analgesia. Experience with BBED in the U.S. has been gained through a multicenter phase 3 clinical trial (DETECT) and an expanded access treatment protocol (NEXT). In this retrospective study, we describe our experience using BBED, with particular focus on pain management.

**Methods:**

A retrospective review was conducted on 29 adult burn patients enrolled in the DETECT or NEXT trials who underwent BBED of acute deep partial- and full-thickness thermal burn wounds at a major burn center between November 2016 and July 2022. Patient demographics and procedural characteristics, including pain management strategies and Numerical Pain Rating Scale (NPRS) scores before, during, and after debridement, were analyzed and described.

**Results:**

Twenty-nine patients with an average age of 41.2 years (SD=17.8, range 18-72) and an average TBSA of 6.3% (SD=5.6, range 1-24.5) underwent a total of 29 BBED. For pain control during debridement, 6 patients required conscious sedation (CS), 7 a regional block (RB), 5 a local block (LB), and 9 only IV and oral (IVPO) medications. Two patients were intubated and sedated prior to the procedure. No patient required additional BBED treatment and all patients achieved >95% eschar removal. The average NPRS pain score 24 hours before treatment, during treatment, and 24 hours after treatment was 4.0, 4.6, & 4.4, respectively (P=0.65). Sixteen patients (55%) healed without the need for autografting. The average number of days from debridement to wound closure for all patients was 34.9 days (SD=11.9) and for patients who underwent autografting was 40.1 days (SD=12.3).

**Conclusions:**

With appropriate analgesia, it is possible to perform BBED of acute deep partial- and full-thickness thermal burns without significant changes in patient-reported pain scores. Prior to debridement, it is critical to establish procedure logistics and reasonable patient expectations.

**Applicability of Research to Practice:**

The authors review their experience controlling pain associated with BBED.

